# The Influence of the Vacuum Impregnation, Beetroot Juice, and Various Drying Methods on Selected Properties of Courgette and Broccoli Snacks

**DOI:** 10.3390/foods12234294

**Published:** 2023-11-28

**Authors:** Magdalena Kręcisz, Bogdan Stępień, Jacek Łyczko, Piotr Kamiński

**Affiliations:** 1 Institute of Agricultural Engineering, Wroclaw University of Environmental and Life Sciences, Chełmońskiego Street 37a, 51-630 Wrocław, Poland; bogdan.stepien@upwr.edu.pl (B.S.); 120613@student.upwr.edu.pl (P.K.); 2 Department of Food Chemistry and Biocatalysis, Wrocław University of Environmental and Life Sciences, 50-375 Wrocław, Poland; jacek.lyczko@upwr.edu.pl

**Keywords:** drying, vacuum impregnation, VOCs, broccoli, courgette, bulk density, shrinkage, color, VGI

## Abstract

The drying process is used in the food industry to extend the shelf life of fruits and vegetables without the use of preservatives. As quality, visual, and aroma characteristics are important determinants of consumer interest, they play a key role in the development of new foods. In the present study, vacuum impregnation (VI) was used prior to vacuum drying (VD) and freeze drying (FD) of courgette and broccoli. Organic beet juice was used to produce the novel snacks. The study showed that the use of vacuum impregnation significantly affected the VOCs profile (volatile organic compounds profile), in which the following compounds were found: viz: 2-(*E*)-hexen-1-ol, 2-(*Z*)-hexen-1-ol and aceto-phenone. VI caused a decrease in volumetric gel index (VGI), drying shrinkage (S), water activity (AW), decreased color saturation (∆C), and increased dry matter content (DM). All these properties testify to the positive effect of the pretreatment used. The drying methods used had a significant effect on the properties of the dried vegetables. The dries obtained by the FD method showed higher density and water activity, as well as better preserved color (lower ∆E) and higher VOCs, so it is considered that freeze drying is a suitable method for obtaining novel courgette and broccoli snacks.

## 1. Introduction

The volume of world vegetable production from 2000 to 2021 increased by 40%. Consumption of vegetables is very important for consumers. A 2021 study found that more than a quarter of the German and Australian populations mainly consume vegetables, fruits, and whole-grain products [1]. Vegetables are an important source of nutraceuticals in the human diet, which are naturally occurring substances in food. Nutraceuticals are important in the daily diet because they benefit the human body, improve physiological functioning, and improve overall health. Nutraceuticals are ingredients such as antioxidants, vitamins, minerals, and natural pigments (e.g., carotenoids) [2].

Broccoli (*Brassica oleracea* L. var. *italica*) is a popular vegetable worldwide. Its largest producers are China and India [3]. In the United States, broccoli consumption per capita in 2022 was 6.51 g per day, and a 2% increase over the previous year was observed [4]. Broccoli has high nutritional value because it contains bioactive compounds (e.g., sulforaphane, glucosinolate, flavonoids (kaempferol, quercetin), carotenoids (lutein)), and nutrients (i.e., dietary fiber, vitamins (A, C, and vitamin B complex), minerals (e.g., selenium, potassium), and amino acids) [3,5]. Phytochemicals and nutrients in broccoli support well-being, reduce inflammation, and prevent cancer. Broccoli is characterized by antioxidant, anti-inflammatory, antimicrobial, and anticancer effects [6]. In addition, broccoli is low in calories, so it can be a healthy, light snack for people who want to maintain a normal weight, lose weight, or just eat healthy [7].

Courgette (*Cucurbita pepo* L.) is a seasonal vegetable that is popular around the world [8]. In Australia in 2021/2022, the annual consumption of courgette was 17.520 tons, equivalent to 680 g/person per day [9]. Courgette is eaten raw, but due to its rapid loss of freshness, it is used for processing. Courgette contains about 93.5–95% water, so it has a very low energy value. Courgette plays an important role in a balanced diet due to its high nutrient content, including carbohydrates, protein, folic acid, fiber, vitamins, and minerals such as potassium, phosphorus, magnesium, and calcium [8,10].

Red beetroot (*Beta vulgaris* L.) is a valuable raw material used in the food industry. It is used to produce frozen, dried, preserved, and fermented food, as well as concentrates and juices [11]. The popularity of this vegetable is related to the content of many valuable nutrients, including vitamins (A, K, E, C, and B vitamins), minerals (potassium, sodium, magnesium, iron, zinc, boron, silicon, copper, selenium, and manganese) [12]. Red beetroot belongs to the group of health-promoting foods due to the content of the above-mentioned nutritional compounds and bioactive ingredients, i.e., phenols, betalains, and inorganic nitrates [13]. Betalanins, substances contained in beetroot, are used as natural dyes in food production. They are an interesting alternative to artificial dyes. Firstly, because of the possibility of changing the color of food products in a natural way, and secondly, because of the possible health benefits for humans [14]. Based on literature data, red beetroot has strong antioxidant, anti-inflammatory, anti-stress, anti-hypertensive, anti-viral, anti-obesity, anti-cancer, and anti-bacterial properties, as well as lowering lipid levels [15,16]. All the health-promoting properties discussed confirm that red beetroot has great potential as a functional food ingredient. Most importantly, it is used in industry as a raw material for the extraction of a natural food additive, thanks to which it is possible to use beetroot juice to produce innovative, health-promoting food [17].

The development of techniques to extend the shelf life of fruits and vegetables is increasingly driving the food market [18]. Most vegetables and fruits are sourced seasonally, and, due to the high cost of storing fresh materials, they brown, lose firmness and rot quite quickly. This is why proper preservation of fruits and vegetables is so important. Some of the most important methods of food preservation are dehydration and drying. These techniques are commonly used not only to preserve food but also because of the reduction in weight for transportation and the smaller volume of the product, which is important for packaging [2,8]. Processed products, on the other hand, may show changes in sensory, functional, nutritional, and physicochemical properties, especially those that are more sensitive to changes in light, pH, or heat [19].

Technological processes such as osmotic dehydration and vacuum impregnation can help produce products rich in bioactive and volatile compounds [19,20,21,22]. The processes involved in the osmotic drying of fruits and vegetables are now widely studied, as evidenced by recent research papers [22,23,24,25]. Osmosis involves the transport of water from the interior of the sample into a hypertonic solution, with the simultaneous transport of osmotic matter into the interior of the sample, resulting in the leaching of natural solutes [26].

Vacuum impregnation is a variation of osmotic dehydration but is carried out under reduced pressure [20]. Vegetable tissues are well suited for the application of the vacuum impregnation process because their interior is filled with pores containing fluids and air [27]. Depending on the type and composition of the impregnation solution used to VI vegetables and fruits, different results could be obtained. Literature reports provide examples of changing physicochemical properties, protecting against browning, increasing antioxidant properties, bioactive properties, and volatile compounds, and affecting texture through the use of appropriate impregnating solutions [21,28].

In addition, the subsequent use of vacuum drying or freeze drying can favorably extend the storage time and shelf life of fruits and vegetables. This is due to the reduction in water content, which thus makes it possible to inhibit the growth of microorganisms and expand the range of ready-to-eat products offered [20]. Freeze drying is considered one of the best methods for removing water from plant materials. This technique makes it possible to obtain drought of the highest nutritional and sensory quality [29,30,31].

Creating healthy and practical vegetable-based products is an ongoing challenge in the food industry. The purpose of this study was to produce novel dried courgette and broccoli snacks using vacuum impregnation as a pretreatment method. In addition, organic beet juice was used as an impregnating substance aimed at improving the properties of the vegetables. Then two drying methods (vacuum drying (VD) and freeze drying (FD)) were compared and their effects on physical and chemical properties, i.e., dry weight, water activity, gelation index, shrinkage, density, color, and VOCs.

## 2. Materials and Methods

### 2.1. Reagents and Standards

All reagents and organic solvents were of analytical grade and mass spectrometry purity. Undecan-2-one and cyclohexane were obtained from Sigma-Aldrich (Steinheim, Germany).

### 2.2. Materials

**Figure 1:**
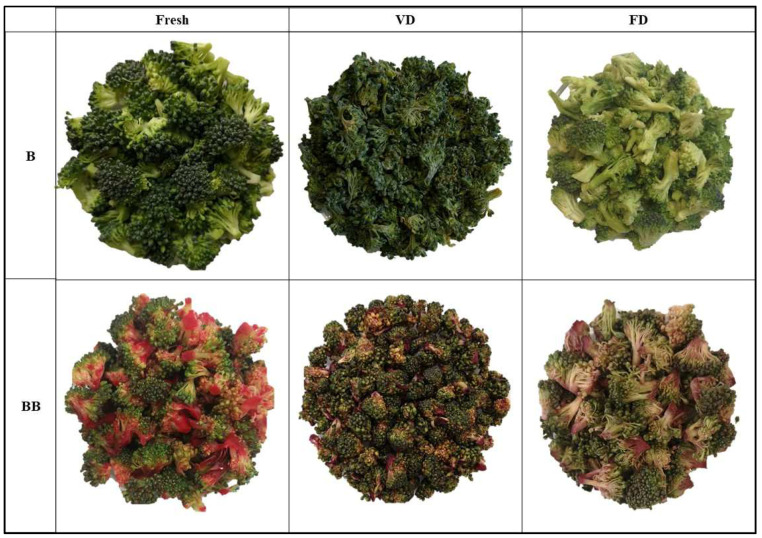
Control sample: fresh broccoli (B), dried broccoli by vacuum drying (VD), freeze drying (FD), broccoli after VI with beetroot juice (BB).

**Figure 2:**
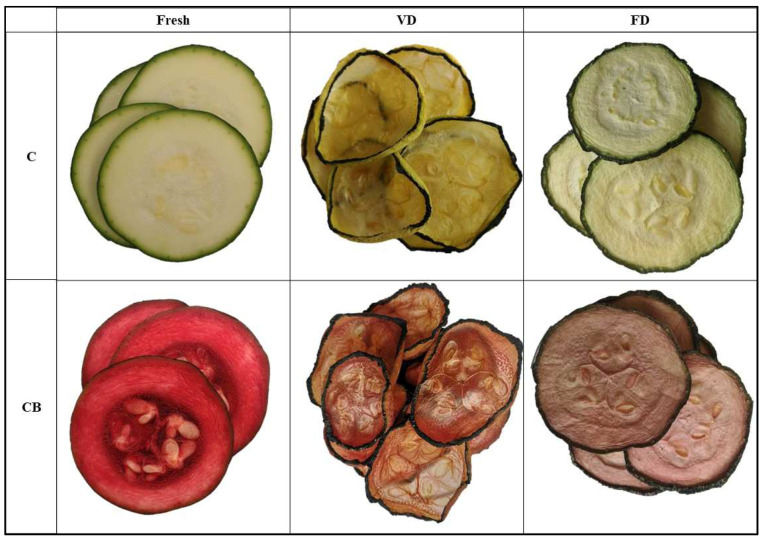
Control sample: fresh courgette (C), dried courgette by vacuum drying (VD), freeze drying (FD), courgette after VI with beetroot juice (CB).

The study material consisted of broccoli and courgette purchased from the local market (commercial products from Lower Silesia, Poland). Before the study, the raw materials were stored in an RL58GRGIH refrigerator (Samsung Electronics Poland, Wronki, Poland) at a constant temperature of 4 ± 2 °C. The vegetables were washed and dried. Broccoli was divided into florets of equal size (approximately 2 cm high and 1.5 cm in diameter), and courgette was cut into slices 5 mm ± 0.1 mm thick, and then subjected to the planned tests. The research used organic, cold-pressed red beet juice. Nutritional value in 100 mL of juice was as follows: Energy value: 155 kJ/37 kcal, fat: <0.5 g, including saturated acids: <0.1 g, carbohydrates: 0.8 g, including sugars: 8.0 g, fiber: <0.5 g, protein: <0.5 g, salt: <0.13 g, potassium: 280 mg (Haus Rabenhorst, Unkel, Germany). Explanations of code designations are given in Table 1, while visualization of all tests is shown in Figure 1 and Figure 2.

### 2.3. Vacuum Impregnation (VI)

Vacuum impregnation was performed in a prototype plant located at the Institute of Agricultural Engineering, Wroclaw University of Life Sciences (Wroclaw, Poland) [20]. The vacuum (VI) impregnation process was carried out at a reduced pressure of 6 kPa and lasted 21 min. A perforated stainless steel vessel with 100 g samples was placed in the impregnation chamber and subjected to reduced pressure for 2 min. Then 1000 mL of impregnating solution was added, and the material was kept in the chamber for 4 min. After restoring atmospheric pressure, the material was kept in the chamber for 15 min. The material was drained using filter paper. As an impregnating solution, organic, cold-pressed red beet juice was used at a concentration of 10.1° Brix (Haus Rabenhorst, Unkel, Germany).

### 2.4. Drying Methods

Two drying methods (vacuum drying (VD) and freeze drying (FD)) were used. For each version of the study, 100 g samples of fresh and vacuum-impregnated broccoli and courgette were used.

#### 2.4.1. Vacuum Drying (VD)

Vacuum drying was carried out at 60 °C, under a vacuum of 10 kPa, in a vacuum laboratory dryer (Memmert, VO101, Schwabach, Germany). The drying time was 24 h.

#### 2.4.2. Freeze Drying (FD)

Freeze drying was carried out at a hotplate temperature of 22 °C under a reduced pressure of 5 Pa, for 24 h in a 4.5 l Free-Zone system (Labconco, Fort Scott, KS, USA). Samples were frozen at −20 °C at a rate of 1 °C/min. A contact method was used to deliver heat to the dried material.

### 2.5. VOCs Extraction and Analysis

VOCs that were released from the samples to the atmosphere headspace solid-phase microextraction Arrow (HS-SPME Arrow) were used. As sorbent, 1.10 mm DVB/C-WR/PDMS SPME Arrow fiber (Shimadzu, Kyoto, Japan) was chosen. Briefly, 0.5 g ± 0.005 of fresh samples or 0.25 g ± 0.005 of dried samples was weighed. Before the extraction, 0.5 µg of undecane-2-one (Sigma-Aldrich, Steinheim, Germany) was used as an internal standard. The extraction was performed in 20 mL headspace vials, which were pre-conditioned at 45 °C for 5 min; then the VOCs were extracted for 30 min at the same temperature, while the sample was shaken at 250 rpm. Thereafter, the analytes were thermally desorbed for 3 min at the injection port temperature. The analyses were run in triplicate.

The separation, identification, and quantification of analytes were performed on the Shimadzu QP 2020 Plus apparatus (Shimadzu, Kyoto, Japan) equipped with a ZB-5Msi (Phenomenex, Torrance, CA, USA) column (30 m × 0.25 mm × 0.25 µm). Injector conditions: Temperature 220 °C; helium as carrier gas with linear velocity 35.0 cm·s^−1^; split 10. The analyte separation was carried out with the following temperature program: 40 °C, then 130 °C at a rate of 3 °C·min^−1^; then 280 °C at a rate of 20 °C·min^−1^, held for 5.5 min. The MS mode was scanned (40–400 *m*/*z*) with an ion source temperature of 250 °C and an interface temperature of 250 °C.

The analyte identification was based on the comparison of linear retention indices (LRIs) and mass spectra. As a reference database, the Flavours and Fragrances of Natural and Synthetic Compounds 3.0 (FFNSC 3.0) library was used. The experimental LRIs were calculated on the basis of simultaneous analysis of the n-alkanes c 7-c 40 mixture (Sigma-Aldrich, Steinheim, Germany). The identification limits were as follows: LRIs ±15; mass spectra similarity ≥90%. Area normalization with an internal standard peak area was used as the quantification method.

### 2.6. Physical Properties

#### 2.6.1. Dry Matter (DM)

Broccoli and courgette samples weighing 0.5 g were measured using an electronic balance (AS160/C/2, Radwag, Radom, Poland; accuracy of measurement: ±0.0001 g). The fresh and dried samples prepared in this way were dried at 70 °C at 3 kPa for 24 h. A vacuum dryer V0101 (Memmert, Schwabach, Germany) was used for the tests [32].

#### 2.6.2. Water Activity (AW)

An AquaLab 4TE ± 0.003 apparatus (AquaLab, Warsaw, Poland) was used to measure water activity. The tests were performed at a constant temperature (25 °C) according to the manufacturer’s instructions. The result is the average of three measurements.

#### 2.6.3. Bulk Density (*ρ_b_*)

Bulk density was performed using a measuring cylinder filled with material [33]. The measurement was made in triplicate and calculated using the formula:

(1) $$\rho_{b}=\frac{w_{s}}{V}$$

gdzie:

*ρ_b_*_—_bulk density [kg · m^−3^],
*w_s_*—weight of samples [kg],
*V*—volume [m^3^].

#### 2.6.4. Volumetric Gel Index (*VGI*)

Volumetric gel index (*VGI*) was determined according to the method developed by Kim et al. [34] with our own modifications. Two malletiers of crushed sample was suspended in 20 mL of distilled water at 20 °C in a measuring cylinder and stirred over a 2 min period. The solution was allowed to swell for 15 min, and then the gel volume was noted. The volumetric gel index of the samples was calculated from the formula:

(2) $$VGI=\frac{V_{g}}{V_{t}}$$

gdzie:

*VGI*—volumetric gel index [%],
*V_g_*—volume of gel [mL],
*V_t_*—sample volume [mL].

#### 2.6.5. Shrinkage (*S*)

Drying shrinkage (*S*) was determined as the ratio of the volume of material after drying (*V_k_*) to the volume of material before drying (*V*_0_) according to the given formula [35]:

(3) $$S=\left(1-\frac{V_{k}}{V_{0}}\right)\cdot 100\%$$

#### 2.6.6. Color

The Color Minolta Chroma Meter CR-200 colorimeter (Minolta Corp., Osaka, Japan) was used to measure the color. A color measurement was performed using a colorimeter with a measuring aperture of 0.008 m in diameter. The CIE-Lab scale was used to evaluate L* for brightness, a* for (+) redness/(−) greenness, and b* for (+) yellowing/(−) blueness, respectively. A D65 light source and a standard colorimetric observer with a field of view of 10° were used. The result is the average of 10 measurements.

The total color change (∆*E*) between dried and fresh vegetables was calculated according to the equation [36]:

(4) $$∆E=\sqrt{\left[{\left(L_{sample}^{*}-L_{control}^{*}\right)}^{2}+{\left(a_{sample}^{*}-a_{control}^{*}\right)}^{2}+{\left(b_{sample}^{*}-b_{control}^{*}\right)}^{2}\right]}$$

where:

L*_control_, a*_control_, and b*_control_—color of fresh materials,
L*_sample_, a*_sample_, and b*_sample_—color of dried samples.

The color saturation (∆*C*) was calculated according to the equation [37]:

(5) $$∆C=\sqrt{a^{*}+b^{*2}}$$

The browning index (*BI*) was calculated based on the equations (Equations (6) and (7)) provided by Dziki et al. [37].

(6) $$BI=100\cdot \left(\frac{X-0.31}{0.17}\right)$$

where:

(7) $$X=\frac{a^{*}+1.75L^{*}}{5.645L^{*}+a^{*}-3.012b^{*}}$$

### 2.7. Statistical Analysis

Statistical analyses, multiple regression equations, determination, and correlation coefficients were performed using Statistica version 13.1 (StatSoft, Tulsa, OK, USA). The results were presented as the mean ± standard deviation. Bidirectional analysis of variance (ANOVA) was performed in this study. HSD Tukey’s least significance test (*p* < 0.05) was used to determine homogenous groups. For VOCs before the creation of the heatmaps, the numerical data were standardized by software algorithms. The results were expressed as mean values (n = 3 ± 10) ± standard deviation.

## 3. Results

### 3.1. VOCs Extraction and Analysis

The impregnation procedure was performed on broccoli and courgette, which were thereafter subjected to various drying methods. In the source objects—raw broccoli and raw courgetti—by the HS-SPME technique followed by GC-MS analysis, 56 and 52 VOCs were found, respectively. The whole profiles of VOCs in the samples are presented in Table 2 and Table 3.

In the case of fresh broccoli, the largest proportions of volatile compounds were: 2-(*Z*)-hexen-1-ol (19%), undecane (16%), and 2-(*E*)-hexen-1-ol (11%). The compounds (especially 3) are characterized by an odor similar to green apple and bitter almond. Whereas in fresh courgette: 2-dodecanone (35%), 2-(*E*)-heptenal (34%), and nonan-4-one (6%).

**Figure 3:**
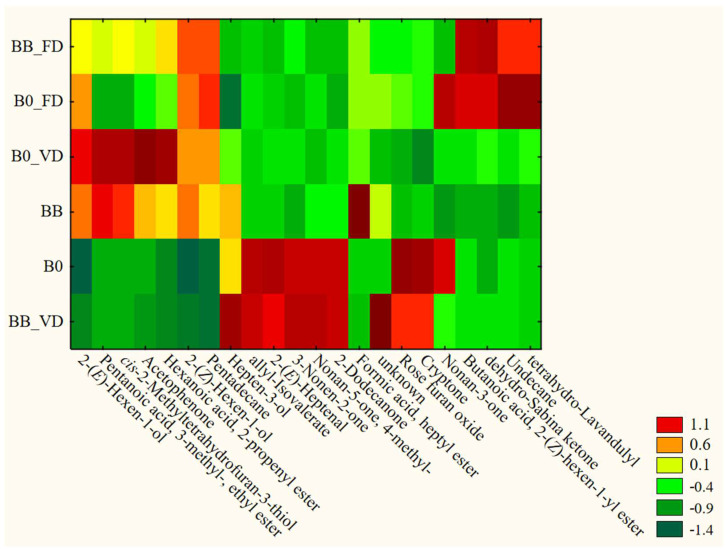
Changes in the VOCs profile of processed broccoli.

On the basis of our earlier research [21,28], it was assumed that the application of impregnation with beetroot juice would significantly change the VOCs profile of the samples. Meanwhile, the examples of broccoli and courgette show that the issue of impregnation is more complex in light of VOCs profile. The broccoli treatment and analysis show that applied drying procedures had a more significant influence on the VOCs profile than impregnation. For instance, the application of freeze drying (FD) has significantly increased the contribution of compounds such as nonan-3-one, 2-(*Z*)-hexen-1-yl ester of butanoic acid, dehydro-sabina ketone, undecane, or tetrahydro-lavandulyl. Meanwhile, vacuum drying was more favorable for 2-(*E*)-hexen-1-ol, 2-(*Z*)-hexen-1-ol, 2-(*E*)-heptrnal, 3-nonen-2-one, or allyl-isovalerate, among others. The heatmap in Figure 3 shows that raw materials (broccoli and impregnated broccoli) that were not dried have different profiles. Impregnations have been introduced into the sample, such as 2-(*E*)-hexen-1-ol, 2-(*Z*)-hexen-1-ol, or acetophenone, while the groups of ketones (e.g., 3-nonen-2-on) were covered down. Nonetheless, after drying, the profiles between broccoli and impregnated broccoli were more similar.

**Figure 4:**
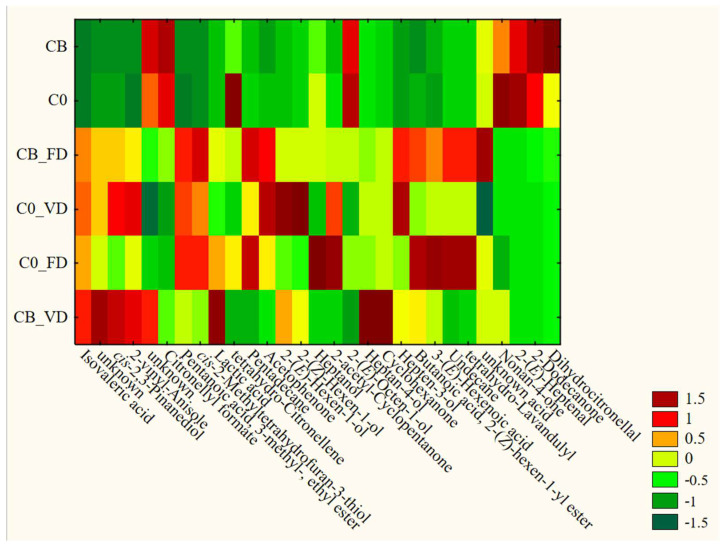
Changes in the VOCs profile of processed courgetti.

The courgette example showed different trends (Figure 4). While raw courgette and raw impregnated courgette showed similar VOCs profiles, after drying, the differences were more significant. Nonetheless, the drying procedure seemed to be more significant than the impregnation procedure for the VOCs profile. The patterns and VOC examples characteristic of FD and VD were similar to those for broccoli treatment. This interesting observation shows that following the impregnation procedure with drying has a different effect on the VOCs profile, which may be related to the source material structure and morphology. The investigation of detailed trends and expectations will require further investigation.

### 3.2. Physical Properties

#### 3.2.1. Dry Matter (DM)

Water is a very important factor affecting food storage. It is believed that water activity in food is a more important physicochemical property than water content because, in heterogeneous systems, it determines the distribution of water between different components [38].

Table 4 shows the results of dry matter content, water activity, and bulk density. No significant effect of the drying method (*p*-value = 0.0443) on dry matter values was observed in the study. Higher DM values were recorded in the material after vacuum drying than after freeze drying. The opposite observations were noted by Kręcisz et al. (2021) when subjecting courgette to vacuum drying (45 °C) [8]. This is likely related to the higher vacuum-drying temperature (60 °C) used in the study reported here [8]. Samples during vacuum drying reach much higher temperatures than during freeze drying. The higher the sample temperature during vacuum drying, the higher the dry matter values. Michalska-Ciechanowska et al. (2021) reported a higher dry weight value for materials vacuum-dried at a higher temperature. Moreover, using inulin and inulin-trehalose, they observed higher water activity and lower moisture content for chokeberry dried in a vacuum at 60 degrees than in the case of freeze-drying [39]. This is related to the different nature of the drying process and the different final temperatures of the material. In the case of freeze drying, the final temperature is 20 °C, and in the case of VD, up to 60 °C. Due to the changes occurring during drying, the products obtained after FD and VD are not the same materials. After vacuum drying, the material reaches an equilibrium moisture level lower than in FD at a much higher temperature, which may also be related to high drying shrinkage, color change, and degradation of volatile compounds, which are related to changes in the physical, chemical, and structural properties that may affect the water activity.

The DM of vacuum-dried broccoli (60 °C; 24 h) was 0.973, while Vega-Galvez et al. (2023) observed lower DM values (0.903) for broccoli dried by the same method at 60 °C for 10.5 h. Increasing the drying time was found to result in higher dry matter values [3]. An increase in dry matter content after pretreatment (VI) was observed in all samples tested. These results are in line with studies by other authors, who applied the vacuum impregnation process to treat courgette [8], sweet potato [21], and kale [40]. Table 5 shows the regression equation describing the dry weight, which depends on the drying method used, the vacuum impregnation process, and the type of vegetable. The model presented explains only 22% of the dependence of the three predicates used.

#### 3.2.2. Water Activity (AW)

Water activity and dry weight are parameters that characterize the persistence of a dried vegetable. Plant materials can be considered effectively dried when the water content is less than or equal to 12%. However, only water activity below 0.6 can protect the product from biochemical changes, physical changes, and, most importantly, the proliferation of microorganisms [41]. In all samples tested, AW was <0.6, indicating microbiological safety. The regression equation explains 95% of the dependence on the drying method used, the vacuum impregnation process, and the type of vegetable (Table 5). The study concludes that AW was significantly influenced by the drying method (*p*-value < 0.0001). Lower values of the studied parameter were observed for FD than for VD, which is consistent with previous studies by the authors of this publication [8,21,28]. This may be related to the influence of the drying method on moisture sorption isotherms. Jebitt et al. (2022) examined sorption isotherms depending on the drying method and parameters. The authors noticed that the equilibrium moisture of 10% corresponds to a water activity of approximately 0.35 for freeze drying at 27 °C and a water activity of 0.51 for vacuum drying at 37 °C [42]. This proves that with different drying techniques and the same equilibrium moisture, water activity may be different. Moreover, the authors noticed that the level of equilibrium moisture tends to decrease in relation to the increase in temperature at a given water activity. Furthermore, Catelam et al. (2011) comparing spray and freeze drying, they noticed that the equilibrium moisture tends to decrease with increasing temperature in a given water activity [43]. This decrease may be due to a reduction in the number of active sites due to chemical and physical changes during drying. Brzezowska et al. (2023) reported similar results for various varieties of blueberry fruits when vacuum dried at different temperatures; droughts with lower moisture were characterized by higher water activity [44].

The composition of vegetables and fruits can be presented as an aqueous solution of low-molecular-weight substances consisting of organic acids, sugars, and salts. Additionally, aqueous solutions of substances contain large particles that are contained in the water-insoluble cellular matrix of macromolecules, mainly carbohydrates, pectin substances, proteins, hemicelluloses, and sometimes also lignin. The pulp tissue of fruits and vegetables contains intracellular air spaces that play a huge role in the perception of structure. The ingredients listed above can interact with water to varying degrees, which is why they have the ability to reduce water vapor pressure. Moreover, the water contained in fruits and vegetables may be related to chemical and enzymatic reactions, oxidative changes, and the content of volatile compounds. The quality and stability of processed vegetables and fruits are influenced by physical and structural changes, i.e., collapse during drying, crystallization, loss of crunchiness, loss of volatile compounds, and other forms of texture degradation [45]. The use of the vacuum impregnation process resulted in lower water activity values in all the samples tested. Cichowska-Bogusz et al. (2020) observed that dried apples subjected to the vacuum impregnation process using xylitol also had lower AW values [25].

#### 3.2.3. Bulk Density (ρb)

Bulk density, which is strongly related to the water content of raw materials, is considered one of the parameters that define the structure of materials. Density is determined by the mass of a sample relative to its volume and is closely related to the volume of pores in the materials in question [46]. In our study, we proved that the use of different drying methods and different vegetables significantly affected the values of bulk density (*p*-value < 0.0001). A higher bulk density was observed for broccoli after the drying process compared to courgette. Dries obtained by the freeze method were characterized by a higher BD value than those that were the product of vacuum drying. Materials after VD are characterized by higher shrinkage and reduced porosity [47]. The use of vacuum impregnation had no significant effect on the parameter studied (*p*-value = 0.0518). Reza Askari et al. also observed an increase in bulk density after pretreatment [48].

#### 3.2.4. Volumetric Gel Index (VGI)

**Figure 5:**
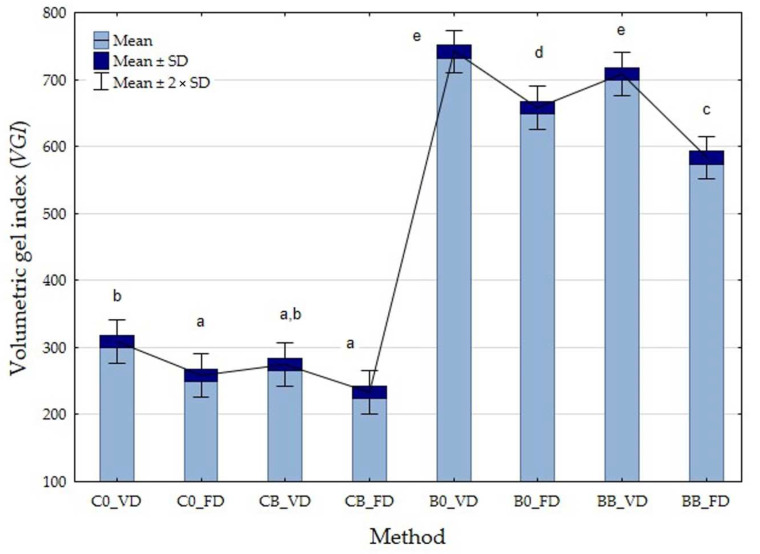
Volumetric gel index of freeze-dried (FD) and vacuum-dried (VD) broccoli (B) and courgette (C) before and after the vacuum impregnation process with beetroot juice. Values followed by the same letter (a–e), were not significantly different (*p* > 0.05), according to Tukey’s HSD test.

More and more dried fruits and vegetables are being added to ready meals, breakfasts, or yogurts, so it is necessary to understand the mechanisms of the dried fruit rehydration process. Material rehydration depends on many factors, such as moisture content, type of pretreatment used, drying conditions, and methods. Thus, the ability of a material to revert to its previous form particularly depends on the internal structure of the material and, thus, on the degree of structural and cellular destruction during the drying process [47]. The displacement of cells and irreversible cracks that occur during the drying process, resulting in a loss of cohesion and, consequently, the dense, collapsed structure and capillary shrinkage that occur, can result in a loss of the ability to absorb the appropriate amount of water necessary for full hydration [49]. The lowest VGI values were observed for CB_FD (233.33%) and the highest values for B0_VD (741.67%). This is probably related to the different structures of these vegetables and the higher water content of courgette. The use of a vacuum impregnation process (*p*-value = 0.0004), different drying methods (*p*-value < 0.0001), and different vegetables (*p*-value = 0.0518) had a significant effect on the value of the gelation index. An average 10% reduction in VGI values was observed in all samples tested after the vacuum impregnation process (Figure 5). The results are consistent with the studied drying shrinkage. Higher absorbency was observed in those materials whose shrinkage was lower. The use of pretreatment prior to the drying process resulted in higher-quality dried material. In the case of the freeze method, the lowest values of the gelation index were observed after vacuum drying. In the case of the freeze method, there is evaporation of water from the frozen material, so the product is characterized by a porous structure, leading to better hydration and a lower gelation index. In the literature, information is available that takes into account the comparison of drying methods in terms of porosity. From this information, it appears that materials with a higher porosity of 80–90% are formed FD [47]. The vacuum method destroys the chambers, making the dried product more difficult to absorb water, and the porosity of the finished product is lower than after FD. The researchers observed that porosity is also related to the type of raw material used. For vacuum drying, porosity was found to be 70% for apples and bananas, 50% for carrots, and 25% for potatoes [47].

#### 3.2.5. Drying Shrinkage (S)

Drying shrinkage occurs during drying. This is when an increase in the force of surface tension occurs, and, as a result, the structure of the material collapses and shrinks. Shrinkage is characterized by a hardened structure occurring both on the surface and in the interior of the dried material [50].

**Figure 6:**
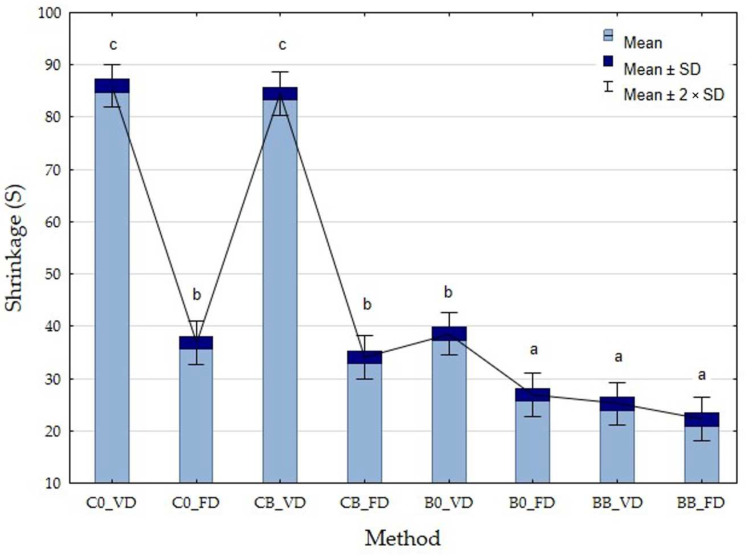
Shrinkage of freeze-dried (FD) and vacuum-dried (VD) broccoli (B) and courgette (C) before and after the vacuum impregnation process with beetroot juice. Values followed by the same letter (a–c), were not significantly different (*p* > 0.05), according to Tukey’s HSD test.

The study showed that both the drying method and the type of vegetable had a significant effect on the value of the parameter studied (*p*-value < 0.0000). Lower values of drying shrinkage were shown for freeze-dried materials due to slow sublimation. Xu et al. (2020) reported similar results, with the highest drying shrinkage observed with VD and the lowest with FD [30]. Obtaining higher values during VD may be due to the pressure difference between the inner and outer parts of the samples, which can also cause compressive stresses due to shrinkage. The application of the vacuum impregnation process reduced the values of the tested parameter in all the samples, but VI did not significantly affect the tested parameter (*p*-value = 0.2698). Lower values of the parameter were observed for broccoli and occurred at 22.17–38.55% depending on the drying and pretreatment method used (Figure 6). Significantly higher shrinkage values were observed for courgette without VI, which may be related to the considerably higher amount of water contained in courgette. Drying shrinkage hinders the re-hydration of the material and negatively affects the quality of the dried material. Therefore, it is important to keep its values as low as possible [49]. The highest values of drying shrinkage were obtained for untreated vacuum-dried courgette (88.89%). At the same time, the use of vacuum impregnation reduced this index from 59.71% to 57.18%, depending on the drying method used. In the case of the convection drying method, the shrinkage rate is 90%, and in the case of microwave-vacuum drying, it is 70% [50]. Sublimation drying is the method by which the lowest values of the studied parameter were obtained. Jałoszyński et al. proved that after microwave-vacuum drying of parsley root, the drying shrinkage was at the level of 72–78% and depended on the microwave power and particle size of the material [50]. Other authors observed a drying shrinkage of 70% when drying kohlrabi using the microwave-vacuum method [35]. Piotrowski and Ignaczak proved that after vacuum drying (5 kPa) at 60 °C, the drying shrinkage of strawberries was 22.84% [51].

### 3.3. The Appearance and Color Parameters of Fresh and Dried Broccoli and Courgette

All the color parameters of fresh and dried courgette and broccoli are shown in Table 6. The color of food products is one of the most important characteristics for assessing quality and food acceptance, and it also influences the choice of products by consumers. It has been shown that when ∆E > 2, the color of the samples changes significantly from the test sample and is visible to any observer [51]. Figure 1 and Figure 2 clearly show the change in color depending on the drying method used, the vacuum impregnation process, and the type of vegetable.

A significant color change was observed in all samples tested. The smallest change (∆E = 4.64) was observed for freeze-dried courgette, and the largest change (∆E = 61.60) was observed for fresh courgette after applying the vacuum impregnation process. A lower ∆E value was observed for freeze-dried materials in all samples, indicating better color retention when this drying method was used. Similar observations were noted by Xu et al. (2020) and Mohammadi et al. (2020), who dried broccoli using various methods, including FD [30,31]. The visual differences between all samples are shown in Figure 1 and Figure 2. It is clear that the VD-dried vegetables change in color and shape. Although the dried fruit obtained by the VD method had lower color change values, no statistically significant differences were found. Other authors include VD among the drying methods that allow better color retention than convection drying methods [30,31]. The use of beet juice during the vacuum impregnation process, as expected, increased ∆E from 20.57 to 91.36%, depending on the type of vegetable and the drying method. The intense increase in color change was due to the intense dark color of the juice used, and the change would have been greater the lighter in color the vegetable was tested. Other authors have also observed that the material after VI is characterized by a darker color [40].

L* values differed significantly depending on the type of material, VI process, and drying method (*p*-value < 0.0000). An increase in L* values was observed for courgette after FD. These results are consistent with our previous studies, in which we investigated the effect of color on courgette [8], celery [21], and sweet potato [28]. In the case of broccoli after VD and FD drying, as well as courgette after VD, there was a decrease in brightness in all samples tested; this indicates pigment degradation and/or chlorophyll degradation and browning reactions after drying (these values are in accordance with the BI given in Table 5). Application of VI significantly affected the brightness value (*p*-value < 0.0000). As expected, beet juice with an intensely dark color significantly reduced the values of the L* parameter. Seraglio et al. (2023) also observed a reduction in pear brightness after VI with berry juice. These results are comparable, indicating the preservation of pigments from dark juice in the fruit and vegetable pores [20]. A decrease in the value of the L* parameter after the VI process was also observed by Kidoń et al. (2023) and [27]. The authors studied the effect of vacuum impregnation on the properties of apples. The most intense shade of green (a* = −6.64–0.90) was recorded in fresh and dried vegetables and after FD. VI, with the use of beet juice, contributed to the increase in the studied parameter, and at the same time, the red hue was shown.

Positive b* values, which indicated the degree of yellow color retention, were shown for all drought variants obtained by both methods. Higher yellowing values were observed in courgette samples after VD, which is related to the light/yellow color of courgette. The application of vacuum impregnation reduced the value of the yellow color in all samples tested. These results are in line with the observations of Pasławska et al. [40], who studied the effect of the vacuum impregnation process on the properties of kale. Kidoń et al. (2023), while studying the effect of VI on the properties of apples, also observed a reduction in the value of the b* parameter after the vacuum impregnation process [27]. The decrease in the b* parameter may be due to an increase in the transparency and vitreousness of the materials, especially evident in courgette after VD (Figure 2).

The application of the vacuum impregnation process reduced the color saturation value ∆C in both broccoli and courgette. These observations are consistent with Pasławska et al. (2018), who studied the effect of the vacuum impregnation process on the properties of kale [40].

## 4. Conclusions

During the study, it was observed that both the addition of beet juice, the vacuum impregnation process, and the use of different drying methods had a significant effect on the physicochemical properties.

The results confirmed that the use of vacuum impregnation as a pretreatment method before the drying process is an effective method for improving the properties of the tested vegetables. The use of VI resulted in lower drying shrinkage, VGI, lower water activity, and dry matter. In addition, the use of beet juice during VI directly influenced the saturation of volatile organic compounds, especially evident in the case of courgette, which was characterized by a softer texture, making it possible to introduce more impregnating substances into its pores.

The results confirmed that dried fruit obtained by the freeze method was characterized by higher water activity, density, VGI, and better color and shape retention compared to the vacuum method. Therefore, this method is recommended for the production of health-promoting foods.

The obtained results of selected physicochemical properties, i.e., color, water activity, shrinkage, density, VOCs, dry weight, and VGI, are important in the context of producing new functional foods. Broccoli and courgette are low-calorie products, so they can successfully provide a healthy and light snack, even for people with a healthy lifestyle. The use of organic beet juice as an impregnant allowed the introduction and identification of 56 VOCs, including 2-(*E*)-hexen-1-ol, 2-(*Z*)-hexen-1-ol, or acetophenone. This research has successfully provided valuable information on the first such courgette and broccoli snacks containing beet juice. However, further research is needed that takes into account the content of bioactive ingredients.

## 5. Patents

Patent Poland, no. 421913. Vacuum impregnating machine and method for initial processing of material. Wrocław University of Environmental and Life Sciences, Wrocław, PL. Authors: Bogdan Stępień, Radosław Maślankowski, Leszek Rydzak, and Marta Pasławska.

## Figures and Tables

**Table 1:** Fresh and dried materials samples codes.

Code	Material	Type of Drying
C0	Courgette	-
C0_FD	Courgette	Freeze drying
C0_VD	Courgette	Vacuum drying
CB	Courgette after impregnation with beetroot juice	-
CB_FD	Courgette after impregnation with beetroot juice	Freeze drying
CB_VD	Courgette after impregnation with beetroot juice	Vacuum drying
B0	Broccoli	-
B0_FD	Broccoli	Freeze drying
B0_VD	Broccoli	Vacuum drying
BB	Broccoli after impregnation with beetroot juice	
BB_FD	Broccoli after impregnation with beetroot juice	Freeze drying
BB_VD	Broccoli after impregnation with beetroot juice	Vacuum drying

**Table 2:** VOCs profile of raw broccoli.

Compound	LRI_exp_ ^1^	LRI_lit_ ^2^	Spectrum Match [%] ^3^	[%]	SD
2-(*E*)-hexen-1-ol	862	864	90	11.03	0.18
2-(*Z*)-hexen-1-ol	865	866	91	19.47	0.60
Hepten-3-ol	870	877	90	6.92	0.22
allyl-isovalerate	924	934	92	1.22	0.06
Pentanoic acid, 3-methyl-, ethyl ester	956	953	95	4.46	0.51
2-(*E*)-heptenal	962	956	91	0.60	0.15
cis-2-methyltetrahydrofuran-3-thiol, cis-	966	956	94	3.38	0.36
Heptanol	971	970	96	0.81	0.09
Dec-1-ene	980	987	90	0.85	0.06
Hexanoic acid	987	990	92	0.54	0.03
Octan-2-one	993	990	90	0.37	0.00
Decane	1001	1000	93	0.43	0.01
Octanal	1003	1006	90	0.14	0.00
2,4-(E,E)-heptadien-1-ol	1012	1019	94	0.34	0.02
Formic acid, heptyl ester	1015	1024	91	1.75	0.09
Heptanoic acid, methyl ester	1025	1025	90	0.14	0.01
isoamyl-pyruvate	1043	1051	91	0.26	0.02
2-(*E*)-octenal	1054	1059	93	0.07	0.00
Acetophenone	1060	1068	95	3.96	0.17
2-(*E*)-octen-1-ol	1070	1067	91	0.27	0.01
Octanol	1074	1076	90	0.27	0.01
Hexanoic acid, 2-propenyl ester	1081	1081	92	1.44	0.24
Nonan-3-one	1087	1088	95	0.42	0.00
Butanoic acid, 2-(*Z*)-hexen-1-yl ester	1089	1099	90	5.30	0.21
Unknown	1091			0.63	0.02
Undecane	1100	1100	94	15.69	0.48
3-octen-2-one, 7-methyl-	1108	1106	90	0.34	0.02
1,3,8-para-menthatriene	1124	1110	92	0.10	0.01
Nonan-5-one, 4-methyl-	1126	1111	95	0.45	0.02
dehydro-Sabina ketone	1128	1122	91	0.72	0.02
Dihydrocitronellal	1130	1125	94	0.14	0.01
Unknown	1133			0.26	0.02
Propanoic acid, cyclohexyl ester	1134	1135	90	0.33	0.02
2-(*E*)-butenoic acid, 2-methyl-, 1-ethyl-2-propen-1-yl ester,	1138	1137	90	0.33	0.00
3-nonen-2-one	1139	1137	94	0.21	0.01
Butanethioic acid, 3-methyl-, S-(1-methylpropyl) ester	1153	1148	96	0.62	0.04
3,6-nonadien-1-ol	1155	1154	91	tr ^4^	0.01
tetrahydro-lavandulyl	1164	1162	90	1.15	0.02
Unknown	1166			0.24	0.04
Rose furan oxide	1171	1169	92	1.54	0.06
Cryptone	1194	1187	90	2.68	0.12
Dodecane	1200	1200	94	0.82	0.01
Decanal	1205	1208	91	0.18	0.02
2-(*E*)-octen-1-ol, acetate	1208	1210	93	0.50	0.02
Ionane	1244	1246	90	0.30	0.00
2-decen-1-ol	1256	1270	92	0.25	0.02
tetrahydro-lavandulyl acetate	1270	1272	93	0.64	0.01
Tridecene	1291	1292	91	0.17	0.02
Undecanal	1311	1309	94	0.13	0.00
cis-2,3-pinanediol	1321	1321	90	0.11	0.00
2-undecen-1-ol	1363	1370	91	0.40	0.03
2-dodecanone	1393	1392	93	1.51	0.14
Tetradecane	1399	1400	95	0.33	0.02
trans-2-dodecen-1-ol	1476	1471	92	0.25	0.03
Pentadecane	1499	1500	96	4.29	0.12
2-(*E*)-tridecen-1-ol	1569	1573	91	0.16	0.00

^1^ Experimentally obtained linear retention index; ^2^ literature (FFNSC) linear retention index; ^3^ similarity between experimentally obtained mass spectra and ones available in database (FFNSC); ^4^ tr < 0.10%.

**Table 3:** VOCs profile of raw courgetti.

Compound	LRI_exp_^1^	LRI_lit_^2^	Spectrum Match [%] ^3^	[%]	SD
Isovaleric acid	849	942	90	0.21	0.12
2-(*E*)-hexen-1-ol	863	864	91	tr ^4^	-
2-(*Z*)-hexen-1-ol	865	866	90	0.34	0.04
Hepten-3-ol	870	877	93	1.23	0.11
Unknown	875			0.46	0.11
Lactic acid	880	877	90	0.31	0.08
Heptan-4-ol	890	891	92	0.35	0.00
5-hydroxy-pentanal	891	889	90	0.14	0.01
unknown acid	899			5.77	0.01
Cyclohexanone	906	901	91	0.55	0.07
Heptanal	908	906	94	0.26	0.05
tetrahydro-citronellene	924	930	90	4.07	0.48
Pentanoic acid, 3-methyl-, ethyl ester	953	953	95	tr	-
cis-2-methyltetrahydrofuran-3-thiol	962	955	91	tr	-
2-(*E*)-heptenal	963	956	93	33.65	2.05
Heptanol	976	970	95	3.12	0.18
Hexanoic acid	985	997	91	tr	-
Decane	1001	1000	94	tr	-
Octanal	1003	1006	92	0.11	0.03
Unknown	1008			tr	-
Acetophenone	1060	1068	96	0.33	0.08
Unknown	1068			tr	-
2-(*E*)-octen-1-ol	1074	1069	92	1.03	0.05
3-(*E*)-hexenoic acid	1080	1071	91	tr	-
Nonan-4-one	1085	1078	93	6.05	1.14
2-acetyl-cyclopentanone	1088	1078	94	0.48	0.48
Butanoic acid, 2-(*Z*)-hexen-1-yl ester	1089	1099	97	0.21	0.00
Undecane	1100	1100	94	0.45	0.00
Nonanal	1104	1107	90	0.17	0.01
Unknown	1124			1.54	0.05
α-Campholenal	1128	1125	95	0.47	0.00
Dihydrocitronellal	1130	1125	92	0.64	0.22
Propanoic acid, cyclohexyl ester	1134	1135	75	0.14	0.09
2-vinyl-anisole	1144	1135	90	tr	-
Neothujol	1159	1153	91	tr	-
tetrahydro-lavandulyl	1163	1162	90	tr	-
Dodecane	1200	1200	94	0.23	0.07
Decanal	1205	1208	90	0.47	0.06
2-(*E*)-octen-1-ol, acetate	1208	1210	91	tr	-
trans-2-hydroxy-pinocamphone	1255	1253	92	tr	-
tetrahydro-lavandulyl acetate	1270	1272	93	tr	-
Citronellyl formate	1273	1275	90	1.07	0.13
Tridec-1-ene	1291	1292	90	tr	-
Undecanal	1306	1309	91	0.29	0.02
Acetic acid, nonyl ester	1312	1313	93	0.06	0.05
cis-2,3-pinanediol	1321	1321	90	0.10	0.02
unknown	1363			tr	-
2-dodecanone	1393	1393	94	34.61	1.29
Tetradecane	1399	1400	92	tr	-
Pentadecane	1499	1500	96	0.57	0.01
2-(*E*)-tridecen-1-ol	1569	1573	91	tr	-
Javanol isomer II	1612	1612	91	tr	-

^1^ Experimentally obtained linear retention index; ^2^ literature (FFNSC) linear retention index; ^3^ similarity between experimentally obtained mass spectra and ones available in database (FFNSC); ^4^ tr < 0.10%.

**Table 4:** Dry matter (DM), water activity (AW), bulk density (ρb).

Method	DM (%)	AW	ρb (kg·m^−3^)
C0	0.071 ± 0.007 ^a^	0.988 ± 0.001 ^f^	250.41 ± 3.66 ^f^
C0_FD	0.956 ± 0.001 ^c^	0.214 ± 0.013 ^b^	14.97 ± 2.61 ^a^
C0_VD	0.969 ± 0.013 ^c^	0.320 ± 0.004 ^f^	41.89 ± 0.10 ^b,c^
CB	0.075 ± 0.001 ^a^	0.985 ± 0.001 ^f^	374.25 ± 6.88 ^h^
CB_FD	0.965 ± 0.002 ^c^	0.159 ± 0.004 ^a^	19.59 ± 3.38 ^a^
CB_VD	0.978 ± 0.012 ^c^	0.308 ± 0.003 ^d,e^	39.94 ± 2.71 ^b^
B0	0.151 ± 0.001 ^b^	0.989 ± 0.002 ^f^	207.47 ± 4.19 ^e^
B0_FD	0.967 ± 0.001 ^c^	0.203 ± 0.011 ^b^	47.78 ± 2.28 ^b,c^
B0_VD	0.973 ± 0.018 ^c^	0.293 ± 0.004 ^d^	90.78 ± 5.58 ^d^
BB	0.154 ± 0.002 ^b^	0.985 ± 0.003 ^f^	341.15 ± 3.25 ^g^
BB_FD	0.969 ± 0.001 ^c^	0.173 ± 0.002 ^a^	49.12 ± 1.30 ^b,c^
BB_VD	0.974 ± 0.003 ^c^	0.273 ± 0.002 ^c^	53.73 ± 3.32 ^b^

Values (mean of three replications) ± standard deviation followed by different letters (a–h), are different (*p* ≤ 0.05) according to Tukey’s test.

**Table 5:** Multiple regression equations, determination, and correlation coefficients.

Tested Value	Regression Equations	R	R^2^
DM	DM = 0.087 + 0.009 · D − 0.004 · M − 0.006 · VI	0.52	0.27
AW	AW = 16.046 − 0.111 · D − 0.015 · M − 0.03 · VI	0.97	0.95
ρb	ρb = 117.825 − 23.719 · D + 31.258 · M − 8.260 · VI	0.91	0.83
VGI	VGI = −28710.417 − 75.000 · D + 404.167 ·M − 41.667 · VI	0.99	0.98
S	S = 6776.251 − 28.582 · D − 32.154 ·M − 5.589 · VI	0.89	0.80
L*	L* = 6845.856 + 6.141 · D − 43.009 ·M − 30.204 · VI	0.88	0.79
a*	a* = 45.943 − 1.221 · D − 6.347 ·M + 7.137 · VI	0.94	0.89
b*	b* = 726.384 + 0.880 · D − 4.986 ·M − 2.974 · VI	0.93	0.87

**Table 6:** Color changes: L*—lightness, a*—greenness, b*—yellowness, BI—browning index, ΔC—saturation, ΔE—total color of vegetables (C—courgette, B—broccoli, 0—without VI) dried by freeze drying (FD) and vacuum drying (VD).

Method	Color					
	L*	a*	b*	BI	∆C	∆E
C0	87.87 ± 1.59 ^h^	−3.59 ± 0.18 ^b^	13.44 ± 0.21 ^i^	13.30 ± 0.40 ^b,c^	13.30 ± 0.21 ^h^	-
C0_FD	91.54 ± 1.9 ^i^	−0.90 ± 0.05 ^d^	12.53 ± 0.28 ^h^	17.14 ± 0.41 ^c,d^	12.62 ± 0.27 ^g^	4.64
C0_VD	85.52 ± 1.17 ^g^	1.68 ± 0.06 ^f,g^	12.55 ± 0.27 ^h^	13.83 ± 0.43 ^b,c^	12.50 ± 0.28 ^g^	5.84
CB	30.70 ± 0.72 ^e^	18.40 ± 0.25 ^j^	6.96 ± 0.33 ^e^	66.37 ± 2.48 ^h^	8.17 ± 0.29 ^e^	61.60
CB_FD	36.09 ± 1.12 ^f^	9.46 ± 0.25 ^h^	7.50 ± 0.33 ^f^	74.14 ± 5.88 ^i^	9.01 ± 0.35 ^f^	53.73
CB_VD	24.75 ± 1.88 ^d^	11.53 ± 0.40 ^i^	8.35 ± 0.38 ^g^	42.03 ± 1.96 ^g^	8.12 ± 0.29 ^e^	65.10
B0	30.62 ± 1.16 ^e^	−6.64 ± 0.55 ^a^	5.70 ± 0.22 ^d^	3.14 ± 1.63 ^a^	5.08 ± 0.25 ^c^	-
B0_FD	19.01 ± 0.61 ^c^	−3.74 ± 0.10 ^b^	7.38 ± 0.38 ^e,f^	20.58 ± 2.95 ^d^	4.18 ± 0.31 ^b^	12.09
B0_VD	16.22 ± 1.04 ^b^	−2.23 ± 0.04 ^c^	4.44 ± 0.29 ^c^	31.42 ± 2.81 ^e^	7.12 ± 0.40 ^d^	15.11
BB	23.39 ± 1.02 ^d^	1.37 ± 0.07 ^f^	1.41 ± 0.16 ^a^	10.49 ± 0.99 ^b^	1.83 ± 0.14 ^a^	11.61
BB_FD	17.53 ± 0.75 ^b,c^	1.82 ± 0.17 ^g^	5.30 ± 0.26 ^d^	37.66 ± 4.07 ^f^	3.93 ± 0.27 ^b^	15.59
BB_VD	13.11 ± 0.82 ^a^	0.55 ± 0.03 ^e^	3.86 ± 0.28 ^b^	43.30 ± 2.46 ^g^	5.47 ± 0.26 ^c^	19.02

Values (mean of three replications) ± standard deviation followed by different letters (a–i), are different (*p* ≤ 0.05) according to Tukey’s test.

## Data Availability

Data are contained within the article.
